# Organoids: their emerging essential role in pathological mechanisms and drug discovery of diabetes and its complications

**DOI:** 10.3389/fphar.2025.1650200

**Published:** 2025-08-25

**Authors:** Xiaoyu Xu, Yunqi Zhang, Yifei Geng, Yun Luo, Xiaobo Sun

**Affiliations:** ^1^ State Key Laboratory for Quality Ensurance and Sustainable Use of Dao-di Herbs, Beijing, China; ^2^ Institute of Medicinal Plant Development, Peking Union Medical College and Chinese Academy of Medical Sciences, Beijing, China; ^3^ Key Laboratory of Bioactive Substances and Resource Utilization of Chinese Herbal Medicine, Ministry of Education, Beijing, China

**Keywords:** diabetes, diabetic complications, organoid, mechanisms research, drug screening, precision treatment

## Abstract

Diabetes mellitus is a metabolic disease with a high global prevalence, which affects blood vessels throughout the entire body. As the disease progresses, it often leads to complications, including diabetic retinopathy and nephropathy. Currently, in addition to traditional cellular and animal models, more and more organoid models have been used in the study of diabetes and have broad application prospects in the field of pharmacological research. We summarized the organoid models that have been developed for the study of diabetes mellitus and its complications, and describe their sources, establishment and maturation measures with a focus on pancreatic organoids. For the first time, we summarized the contribution of organoids in diabetes and its complications in terms of mechanism studies, drug screening, and cellular replacement therapies, in the hope of providing a feasible direction for personalized medicine and precision treatment of diabetes and its complications. In addition, we discuss the strengths and limitations of organoids in the field of diabetes and its complications. Nowadays, people strongly advocate personalized medicine and precision medicine, and in this regard, organoid technology has advantages that are unmatched by any conventional experimental models. By combing organoid technology with high-throughput technologies, “patient-specific” drug screening can be achieved faster and more accurately. Organoids are also becoming a potential source of transplantable tissues and functional cell types for cellular replacement therapies in regenerative medicine. With further development of assembly and vascularization techniques, organoids will gradually mature and improve. In conclusion, the 3D organoid system greatly complements the existing modeling system and may play a significant role in future basic and clinical research.

## 1 Introduction

The history of organoids can be traced back to the 1970s, when primary human skin cells were inoculated with 3T3 cells and the epidermal cells grew from individual cells into colonies, which consisted of keratin-forming cells and eventually formed a stratified squamous epithelium(Rheinwald and Green, 1975; Rheinwatd and Green, 1975). In the 1980s, Bissell et al. found that alveolar-like morphogenesis and the formation of mammary-specific functional differentiation occurred when these cells were cultured on reconstituted basement membranes (Barcellos-hoff et al., 1989; Bissell and Ram, 1989). Sasai eet al. Demonstrated the self-organizing mechanism of local intercellular interactions and found that cortical and retinal neuroepithelial cells still have the potential to build multilayer structures *in vitro* (Eiraku and Sasai, 2012). These examples show that cells have the potential to reassemble and form original organ structures, even if they are completely dissociated.

In this context, the concept of organoids and their application technology emerged. Organoids can be obtained mainly from two types of stem cells: pluripotent embryonic stem (ES) cells and their synthetic induced pluripotent stem cell (iPSC) counterparts, and organ-restricted adult stem cells (ASCs). These are often collectively termed as pluripotent stem cells (PSCs) (Clevers, 2016). Currently, PSCs have been induced to give rise to intestinal, kidney, brain, and retinal organoids, as well as liver tissue. What’s more, both brain organoids and retinal organoids (ROs) have shown properties that can recapitulate human organ development, which is not observed in conventional animal models (Lancaster and Knoblich, 2014). Of course, some conditions need to be met for organoids. For example, they must contain multiple cell types of the organs they model and exhibit some function specific to those organs. It is also essential that the organization of the cells be similar to that of the primary organ itself (Lancaster and Knoblich, 2014).

As a technology that is gaining momentum, there are numerous examples which certify the great strengths of organoid technology. They include: the evaluation of drug toxicity; the study of the early stages of disease onset and organ development; and the study of human diseases, such as cancer, rare genetic diseases, and complex multifactorial diseases (Lancaster and Knoblich, 2014; Skardal et al., 2020; Bock et al., 2021). The three-dimensional (3D) structure of cultured cells improves their experimental accessibility compared with traditional animal-only models; moreover, it reduces the number of animals used according to the “3 R” principle (Flecknell, 2002). The significant advantage of organoids over conventional cell cultures is their ability to mimic disease pathology at the organ level (Rossi et al., 2018; Bock et al., 2021). Concurrently, human organoids hold tremendous potential for drug development and precision medicine; furthermore, they provide tractable *in vitro* models that reveal the complex environment of cells (Rossi et al., 2018; Bock et al., 2021). In addition, they hold the promise of contributing to the field of regenerative medicine and cellular replacement therapy by producing transplantable biological structures (Lancaster and Knoblich, 2014; Clevers, 2016; Rossi et al., 2018). Unlike current organ transplants, this treatment avoids immune rejection (Lancaster and Knoblich, 2014). With the development of various modern technologies, organoid methods coupled with single-cell sequencing and spatial profiling technologies may remedy their deficiencies and expand their applications (Bock et al., 2021).

Diabetes mellitus is a metabolic disease characterized by hyperglycemia with inadequate insulin secretion or resistance. Consequently, the prolonged presence of hyperglycemia leads to various tissue dysfunctions, which in turn cause complications such as diabetic retinopathy (DR) and diabetic nephropathy (DN) (Kautzky-Willer et al., 2016; American Diabetes Association, 2020; Ceriello and Prattichizzo, 2021). Therefore, diabetes mellitus is a long-term metabolic disease with multi-organ involvement. Organoids have been used in the study of diabetes and its microangiopathy, and both exposure to hyperglycemia and inflammatory factors *in vitro* and exposure to a diabetic environment in mice resulted in microangiopathy, including thickening of the basement membranes, decreased endothelial to pericyte ratios, and the upregulation of genes such as Angiopoietin 226, Apelin 25, and TNFRSF11B in diabetic organoids (Wimmer et al., 2019b). Hence, the emergence of various organoids provides a usable platform for the study of diabetes. Therefore, we aimed to summarize the organoid models that can be used in the study of diabetes and its complications and describe the applications of these organoids in disease modeling, mechanism studies, and drug screening, as well as their potential for clinical therapeutic applications. Finally, we discuss the shortcomings of organoid technology in current studies of diabetes and its complications, and provide feasible directions for subsequent pharmacological studies and clinical translation. I searched the PubMed database using the keywords “diabetes,” “diabetic complications,” “diabetic retinopathy,” “Diabetic nephropathy,” “diabetic kidney disease” in combination with “organoid”. We further verify the collected articles to ensure that they are relevant to our topic.

## 2 Organoid models for diabetes

**FIGURE 1:**
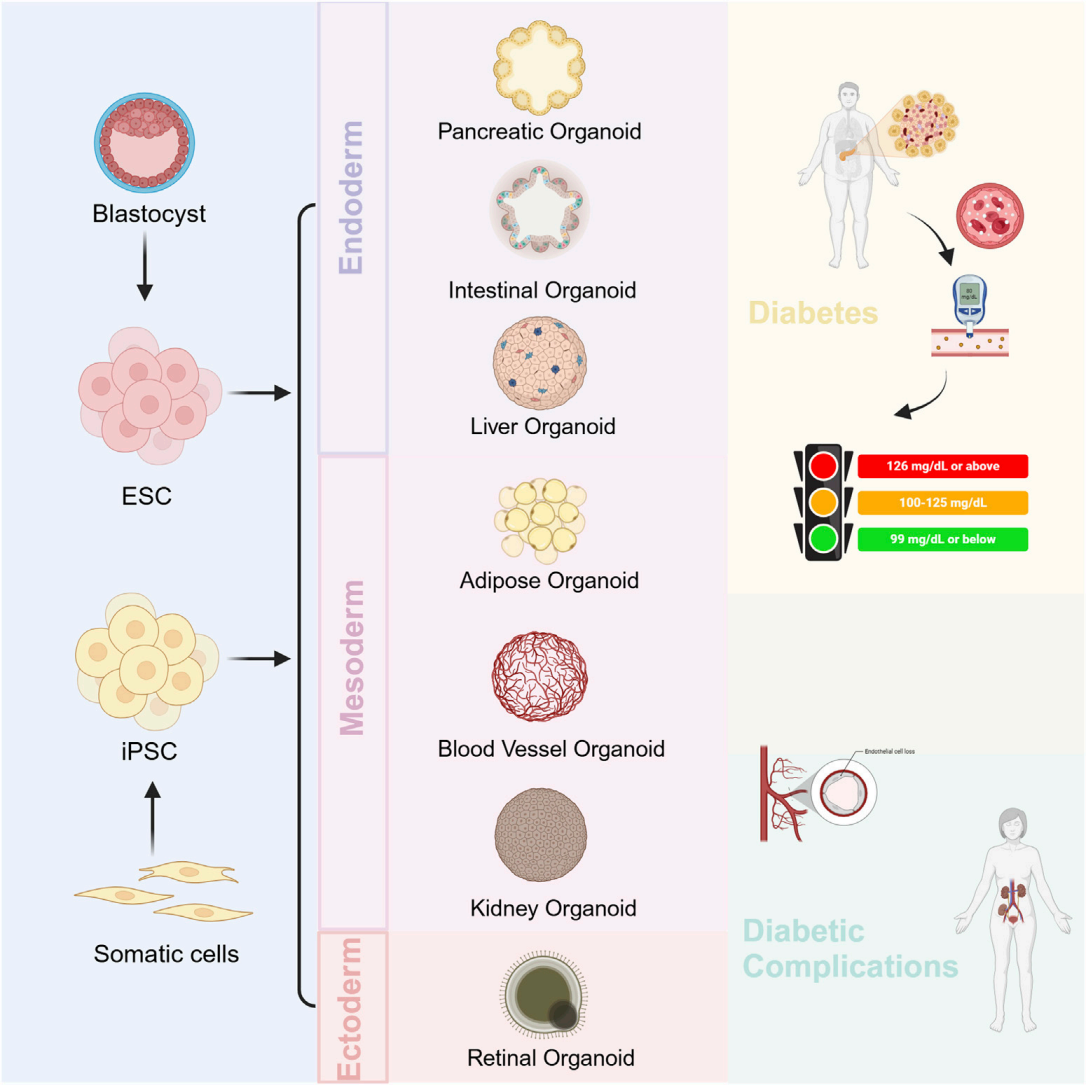
Organoids for the study of diabetes mellitus and its complications.

Diabetes mellitus is a metabolic disease in which multiple organs are involved. Currently, more and more types of organoids are being used in the study of diabetes mellitus and its complications, which we will describe in turn (Figure 1).

### 2.1 Pancreatic organoids

**FIGURE 2:**
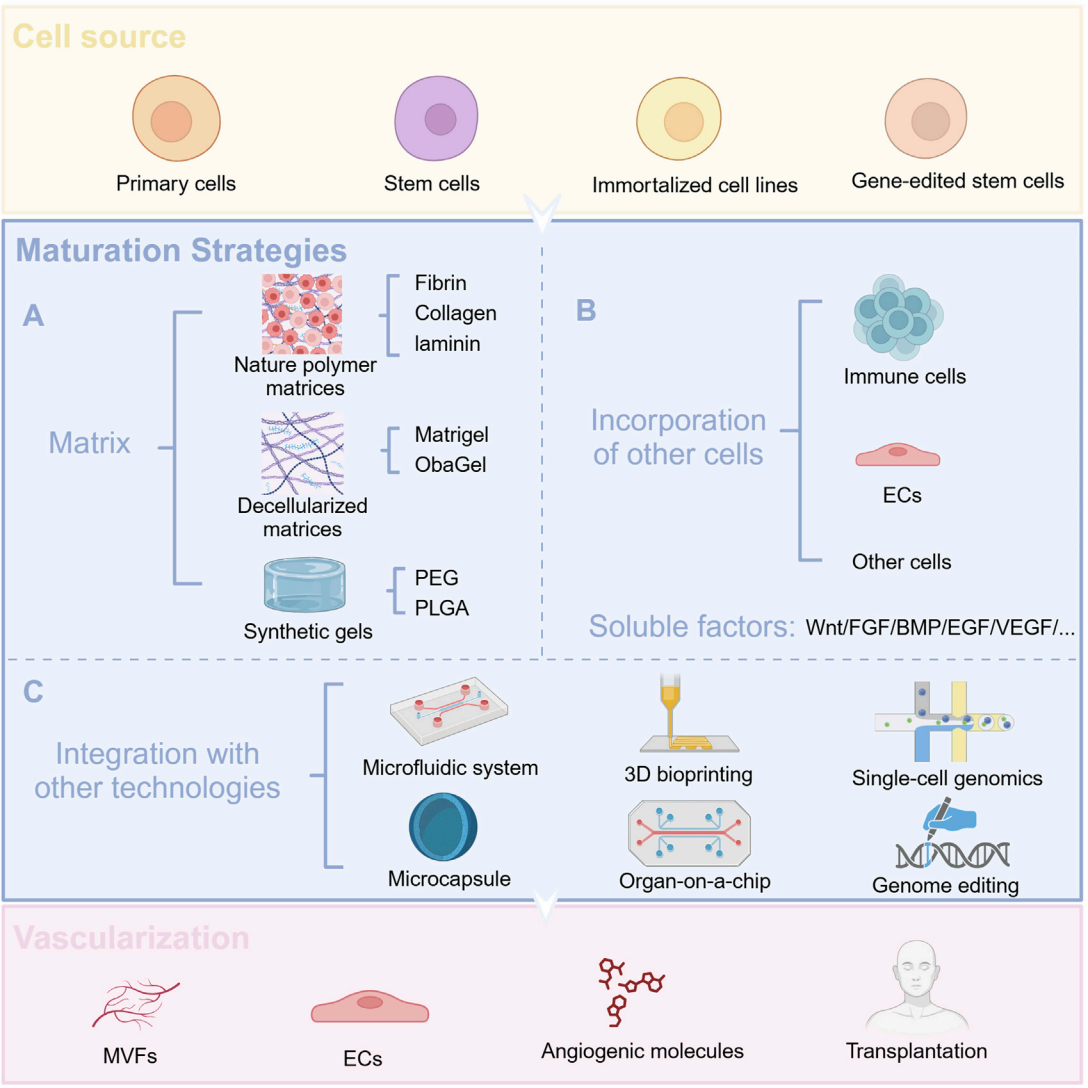
Cellular sources of organoids and optimization strategies. **(A)** Use different matrix gels to promote organoid maturation **(B)** Co-culture with other cells and add appropriate soluble factors to facilitate organoid maturation **(C)** Combine organoid technology with other advanced technologies.

The development and function of the pancreas undoubtedly play a pivotal role in the development of diabetes. Therefore, we have summarized the development of pancreatic organoids in terms of origin, establishment, optimization, and maturation (Figure 2).

#### 2.1.1 Origin and establishment of organoids

Pancreatic organoids arise primarily from two species: mice and humans. Dissociated cell cultures from embryonic, fetal, or adult mouse pancreas can develop to form pancreatic organoids (Grapin-Botton and Kim, 2022). Human fetal cells, adult pancreatic tissue, and PSCs are also gradually being used to construct 3D organ tissues (Loomans et al., 2018; Grapin-Botton and Kim, 2022). With the gradual understanding of the mechanisms of pancreatic islet development, it was realized that cell fate could be designated by the pairing of various combinations of growth factors and small molecules (Zhang et al., 2022). Pagliuca et al. first generated high levels of NKX6.1+/PDX1+ co-expressing pancreatic progenitor cell clusters with the FGF family member KGF, the hedgehog inhibitor SANT1, and retinoic acid. Then attempted combinations of multiple signaling factors including wnt, activin, hedgehog, epidermal growth factor (EGF), transforming growth factor β (TGFβ), thyroid hormones, retinoic acid, and gamma secretase inhibition. Ultimately, human pluripotent stem cells (hPSCs) were successfully utilized to generate hundreds of millions of glucose-responsive β-cells, which were similar to primary β-cells and had an ultrastructure similar to that of adult β-cells (Pagliuca et al., 2014). Similar combinations and selection schemes have been shown in other reports of induction of functional β-cells as well (Nostro et al., 2015; Russ et al., 2015). Notably, the generation of ductal, acinar, and endocrine cells is inextricably linked to NKX6-1 expression, which is an important turning point in the differentiation of hPSCs into pancreatic β-cells. It has also been reported that the transcriptional repressor REST is an important endocrine regulator during pancreatic development and can affect the expression of endocrine genes or the formation of endocrine cells (Rovira et al., 2021). However, the addition of various induction factors should be appropriate, and may be influenced by the cell type selected. For example, there may be a dual-sided or cell-specific role for noggin in the culture of pancreatic islet-like organoids, where, on the one hand, it promotes the differentiation of progenitor cells but, at the same time, it may affect the expression of NKX6.1(Russ et al., 2015; Wesolowska-Andersen et al., 2020). Vitamin C has a similar role: on the one hand, it seems to promote sc-β cell maturation by inhibiting the premature expression of NGN3; however, the cells induced thereby lack glucose responsiveness (Rezania et al., 2014). Hence, we found that the evaluation of pancreatic islet organoids tends to focus on the expression of INS, GLUT2, MAFA, and NKX6.1/PDX1, as well as β-cellular hormone-specific INS genes, expression of C-peptide proteins, glucose-stimulated insulin secretion (GSIS), and higher calcium ion (Ca2+) fluxes.

#### 2.1.2 Maturation strategies for organoids

In addition to being related to the cells used, induction time, cell density, inducer species and ratios, some dynamic culture modes, such as perfused 3D culture conditions, also promote organoid maturation and exhibit enhanced expression of PDX1 and NKX6.1. For example, Tingting Tao et al. used an organ-on-a-chip platform to create a human islet organoid microsystem containing a microfluidic device, which exhibited superior islet organoid differentiation and maturation compared with static culture (Tao et al., 2019). A number of microphysiological systems have also been developed, and studies have shown that such dynamic cultures are more suitable for organoid cultures than static ones (Patel et al., 2021). Furthermore, Carla A Gonçalves found that hPSC-derived pancreatic progenitor cells cultured in 3D were transcriptionally closer to the fetal pancreas, were not dependent on EGF supplementation and had the ability to expand and differentiate (Gonçalves et al., 2021).

The option of biomaterials is an essential step in driving the discovery of organoid technology and the application of regenerative medicine (Noro et al., 2024). Moreover, breakthroughs in materials technology and their integration with stem cell technology will also drive the maturation of organoid technology and contribute to more sophisticated experimental research and broader clinical applications (Liu et al., 2019). Matrigel, derived from Engelbreth-Holm-Swarm mouse sarcoma cells, is a common culture for organoids; however, there are problems with batch variation and safety, and the composition and proportion of Matrigel cannot be accurately determined, which limits the utility of organoids in drug development and regenerative medicine. In view of this, several Matrigel-free culture methods have also been developed, including decellularized ECM, synthetic hydrogels, and gel formation of recombinant proteins (Kozlowski et al., 2021). Hydrogels with high water content have the outstanding advantage of high biocompatibility and can mimic the microenvironment of the natural extracellular matrix (ECM) by modulating their biochemical and physical properties to guide a range of cellular behaviors including cell adhesion, proliferation, migration, differentiation, and cell-cell/cell-matrix interactions in a 3D model (Liu et al., 2019). Hydrogels are further classified into natural and synthetic hydrogel. Natural hydrogels contain bioactive matrices with abundant cell adhesion sites and bionic scaffolds with the ability to encapsulate cells *in situ*, which is favorable for the reconstruction of organoid models *in vitro* (Liu et al., 2019). In contrast, synthetic hydrogels can be individually tailored in terms of composition and mechanical properties to enable more stable and excellent conditions for organoid formation, promote high reproducibility and organoid maturity, and can facilitate the formation of specific tissue/organ models (Liu et al., 2019). Joseph Candiello et al. utilized pancreatic progenitor cells derived from human ES cells and a novel hydrogel system, Amikagel, to generate regenerated islet organoids exhibiting cellular heterogeneity. Their findings demonstrated that Amikagel-induced globules exhibited expression of Pdx-1, NKX6.1, and INS1 genes and augmented C-peptide protein expression (Candiello et al., 2018). Deliang Zhu et al. also found that collagen VI is a key component for normal islet development, and their invention of a Col-VI-based biomimetic ECM can optimize the cell composition and endocrine function of islet organoids (Zhu et al., 2025).

#### 2.1.3 Strategies for vascularization of organoids

Vascularization of islets in clinical transplantation therapy is also a pressing issue. The co-culture of cell lines, natural tissue fragments, and iPSC spheroids with human umbilical vein endothelial cells (HUVECs) and mesenchymal stem cells (MSC) in matrix gel has been used as one of the strategies to vascularize organoids. Human and mouse pancreatic islets with endothelial cells were able to self-organize in a spatiotemporal manner, and such vascularized islets were more effective in treating diabetic mice after transplantation (Takahashi et al., 2018). A polyethylene glycol-maleimide hydrogel and a protein hydrolysis degradable synthetic hydrogel have also been developed to enhance angiogenesis in transplanted pancreatic islets in a diabetic mouse model (Phelps et al., 2013; Weaver et al., 2017; Weaver et al., 2018). Hydrogel infusion showed significantly higher graft survival and superior glucose regulation properties and intra-islet angiogenesis compared with intravenous infusion of islets (Phelps et al., 2013; Weaver et al., 2017; Weaver et al., 2018). Additionally, a 3D printing technique applying a new tissue-specific bioink developed from pancreatic ECM and hyaluronic acid methacrylate has been applied in promoting the formation of pancreatic organoids and their vascular networks, which is expected to improve the effectiveness of islet transplantation (Wang et al., 2023).

### 2.2 Retinal organoids (ROs)

Retinal disease is a leading cause of visual loss and blindness and is associated with complex pathogenesis such as angiogenesis, inflammation, immune regulation, fiber proliferation, and neurodegeneration (Zhang et al., 2021). DR is one of the common microvascular complications of diabetes mellitus. It can cause severe vascular damage and neuronal impairment of the retina, and even result in loss of vision, which has a huge impact on patients’ lives (Cheung et al., 2010; Antonetti et al., 2021). The retina is a highly complex vascularized tissue containing at least 60 functionally distinct cell types, in which various resident cell types communicate with each other and with cells from the blood and immune system (Chichagova et al., 2019; Zhang et al., 2021). Currently, it is also feasible to generate ROs. ROs exhibit consistency with *in vivo* retinogenesis and retinal morphology and can contain most retinal and neuronal cell types, including optic rod and cone cells, ganglion cells, bipolar cells, horizontal cells, amacrine cells, and Müller cells (Chichagova et al., 2019). ROs have been successfully established in many cases, and usually, the addition of appropriate exogenous factors such as BMP, Wnt, Nodal and Notch pathway inhibitors, some growth factors including insulin-like growth factor 1 (IGF1), bFGF, activin, SHH, and triiodothyronine (T3), and serum can induce cell differentiation and determine retinal progenitor cell (RPC) fate (Chichagova et al., 2019). Researchers have found that Dkk1 and Noggin play an important role in enhancing the differentiation of hESCs and hiPSCs to retinal progenitors and photoreceptor precursors at the early stages of differentiation (Mellough et al., 2012). Moreover, ES cells treated with SFEB, Dkk1, LeftyA, serum, and activin produce neural retinal precursors with the ability to differentiate photoreceptors (Ikeda et al., 2005). IGF1 signaling with retinoic acid and T3 is also important for retinal development (Chichagova et al., 2019). Moreover, researchers found that the addition of 9-cis retinoid acid, rather than the commonly used all-trans retinoic acid, accelerated the differentiation of optic rod photoreceptors in organoid cultures (Kaya et al., 2019).

Concurrently, simple and effective strategies have been created that do not require the addition of extrinsic signaling modulators or the involvement of natural retinal cells (Osakada et al., 2008; Zhong et al., 2014). For example, a two-step xeno-free/feeder-free culture system was developed to enable simple and efficient differentiation of hiPSCs into retinal cells (Reichman et al., 2017). In less than 1 month, walled hiPSCs were able to generate self-forming neural retina-like structures containing RPCs (Reichman et al., 2017).

### 2.3 Kidney organoids

hPSCs can differentiate into pluripotent renal unit progenitor cells that form renal unit-like structures and further differentiate into kidney organoids that can mimic kidney development and injury *in vitro* (Morizane et al., 2015). Moreover, 3D kidney organoids have also been successfully generated from different types of source cells, including adult/fetal kidney tissue and kidney cancer biopsies (Liu et al., 2022). In addition to typical kidney organoids, primary renal tubular epithelial organoids, called tubuloids, can be derived from human kidneys and urine and are capable of mimicking key features of renal units in health and disease states (Schutgens et al., 2019; Stower, 2019). These kidney organoids exhibit basic functions such as tubular reabsorption represented by proximal tubular epithelium-mediated dextran uptake and functional renin secretion (Shankar et al., 2021). Xia Y et al. established a diabetes-like renal organoid using alternating 5 mM and 25 mM glucose every 24 h to mimic glucose level shocks in patients with diabetes and found that diabetic-like renal organoids and patients with diabetes had higher angiotensin-converting enzyme 2 (ACE2) expression levels (Xia et al., 2022). However, there are limitations to the application of renal organoids, for example, currently available protocols do not simulate fully mature kidneys; hence, replication of late-onset diseases using organoid technology is not convincing compared with early-onset diseases. It is also worth mentioning that the potential teratogenicity of organoids that are exposed to high-glucose environments should not be overlooked; therefore, a more robust, complete, and realistic renal vasculature system is urgently required (Nishinakamura, 2019; Woolf, 2019). These limitations are expected to be broken one by one with technological breakthroughs. The formation of vascularized glomeruli can be observed by transplantation of iPS-derived renal unit progenitor cells and concomitant transplantation of mixed aggregates of HUVECs and MSCs, among other modalities (Sharmin et al., 2016). In addition, a dynamic culture device, under *in vitro* flow, can induce renal organoid vascularization and promote further maturation of organoid morphology (Homan et al., 2019). Changing the medium composition ratio, by dynamically adjusting Wnt signaling at different stages of organoid differentiation, can produce vascularized organoids and a thickening of the basement membrane of the distal microvascular network, a phenomenon that is inextricably linked to many microvascular lesion (Low et al., 2019). Moreover, kidney organoids show some angiogenic capacity by intrathecal transplantation in chick embryos or by transplantation in chick embryo chorionic allantoic membranes, and organoid-derived endothelial cells can expand to form perfused capillaries and form a vascular network with host-derived blood vessels (Garreta et al., 2019; Koning et al., 2022).

Differentiation in a fully controlled and physiologically relevant 3D growth environment is essential to improve reproducibility and maturation of organoids. Suitable soft environments can accelerate the differentiation of hPSC-derived kidney organoids - the simulation of the intravitreal chorionic villous allantoic membrane microenvironment *in vitro* using a compliant hydrogel promotes the efficient generation of renal vesicles and unit structures (Garreta et al., 2019). Futhermore, after organoids were cultured in a fully synthetic peptide hydrogel, single-cell RNA sequencing showed that this culture mode could produce more mature organoids with fewer off-target cells (Treacy et al., 2023). In conclusion, the application of organoid technology to the study of DN has become possible; however, technological advances are still urgently neede.

### 2.4 Intestinal organoids

The intestine is an important organ involved in human metabolism. Either adult or embryonic stem cells can be used as a source of intestinal stem cells, either by obtaining them from crypt-containing stem cells or isolating individual Lgr5-expressing ISCs from human or mouse small intestinal or colonic tissues, or by direct differentiation of ESCs or iPSCs into 3D intestinal organoids (Zietek et al., 2015; Rahmani et al., 2019). Major components required for culturing intestinal organoids include Wnt-3a (W), EGF (E), Noggin (N), and Rspondin 1 (R) (Rahmani et al., 2019). Intestinal nutrient transport and sensing are also important in diabetes research, and mouse small intestinal organoids enable the study of nutrient and drug transport, sensing and secretion of enteric insulinotropic hormone, and intracellular signaling processes (Zietek et al., 2015). More importantly, glucagon-like peptide-1 (GLP-1), which is released by enteroendocrine cells in the intestine, plays an important role in insulin secretion, food intake, and intestinal peristalsis by increasing insulin secretion and inhibiting glucagon release, as well as delaying gastric emptying and suppressing appetite (Meier, 2012; Gribble and Reimann, 2021; Gribble and Reimann, 2021). Targeting GLP-1 is now an effective treatment for diabetes. Human ileal organoids designed by CRISPR-Cas9 technology have the ability to label and maintain human L cells, opening avenues for the development of drugs targeting the human enteroendocrine system (Goldspink et al., 2020). An intestinal-pancreatic cell co-culture model with 3D morphology also holds promise for screening GLP-1 analogs and stimulants for the treatment of diabetes (Nguyen et al., 2017). Another aspect of intestinal organoids that can be used for diabetes research and treatment is reflected in their cellular transformation capacity. Intestinal endocrine progenitor cells from mice have the potential to be converted into insulin-secreting cells by inhibiting Foxo one or stimulating the expression of Pdx1, MafA, and Ngn3, and can be used as a complementary source in diabetes transplantation therapy (Bouchi et al., 2014; Chen et al., 2014).

The structure and composition of hydrogels for cultured organoids can also be further optimized to promote the differentiation of various enterocyte cell types (Mulero-Russe and García, 2024). ECM hydrogel of gastrointestinal origin has been showen to be a suitable alternative to matrix gel in gastrointestinal organoid cultures, which mimics the organoid microenvironment *in vivo* and enables long-term passaging culture and transplantation of organoids (Kim et al., 2022). Additionally, hydrogel matrix hardness also regulates the trajectory of organoid differentiation (He et al., 2023). Moreover, the combination of intestinal organoids with micro-engineered microarray technology can further promote intestinal organoid maturation (Workman et al., 2018). In conclusion, intestinal organoids also provide a viable platform for the study of metabolic diseases such as diabetes mellitus.

### 2.5 Adipose organoids

Adipose tissue is a major site of insulin resistance in patients with type 2 diabetes mellitus (T2DM), and brown adipocytes (BAs) have also been recognized as a potential cell source for the treatment of metabolic diseases such as diabetes (Zhang et al., 2020; Hu and Lazar, 2022). Previously, researchers used hPSCs and added specific transcription factors-such as nuclear receptor peroxisome proliferator activated receptor γ (PPARγ), CCAAT-enhancer-binding protein-β (CEBPB), and PR domain containing 16 (PRDM16)-to induce the production of white and brown adipocytes (Ahfeldt et al., 2012). Transplantation of hiPSC-derived adipocytes into mice produces well-vascularized adipose tissue and shows glucose uptake capacity (Guénantin et al., 2017), and transplanted BAs also reduce circulating blood glucose levels in hyperglycemic animals (Zhang et al., 2020). Adipose organoids are mainly formed by self-organization of adipose progenitor cells or hPSC and have a structure and function similar to that of adipose tissue (Beydag-Tasöz et al., 2023). Matrix components such as collagen, hydrogel, and ELP-PEI contribute to organoid formation (Hu and Lazar, 2022). Several scaffold-free methods have also been developed to produce 3D fat spheres that can achieve higher levels of lipocalin (Klingelhutz et al., 2018). Cell-to-cell and cell-to-environment interactions are also gradually being emphasized and modeled in adipose organoids (Taylor et al., 2020). In particular, researchers have developed a number of methods for vascularizing adipose organoids. For example, Muller et al. co-cultured human adipose-derived stem cells (hADSCs) with endothelial cells to form patient-specific vascularized adipose organoids that can secrete leptin and can be connected to the vascular system of host mice after transplantation (Muller et al., 2019). Alternatively, the use of adipose tissue-derived stromal vascular fraction or the adoption of other modalities has been shown to be an adipose organ vascularization strategy (Peirsman et al., 2023; Robledo et al., 2023). The use of the human stromal vascular fraction of white adipose tissue as a source of adipose and endothelial progenitor cells to generate vascularized and functional human beige adipose organoids has been reported in studies (Escudero et al., 2023). Retinoic acid also has the potential to promote the development of adipose blood vessels (Wang and Du, 2023).

### 2.6 Liver organoids

The liver primarily consists of epithelial, stromal, endothelial, and mesenchymal cells and is involved in body metabolism as well as exocrine and endocrine processes (Prior et al., 2019). Therefore, liver organoids can be used to study glucose metabolism and insulin resistance in the liver, as well as non-alcoholic fatty liver disease, which increases the risk of T2DM. Liver organoids have been established from iPSCs, ES cells, adult hepatocytes, and cells of adult tissue origin (Hu et al., 2018; Prior et al., 2019). Additionally, more and more liver organoids with self-renewal capacity and mature functions are being developed for disease modeling, drug screening, and precision medicine (Mun et al., 2019; Shinozawa et al., 2021). Moreover, technologies such as CRISPR-Cas9 can also contribute to this goal (Hendriks et al., 2021). A microfluidic multi-class organ system developed using a 3D co-culture of hiPSC-derived liver and pancreatic organoids has also been developed and exhibits activation of metabolism-related signaling pathways, and an increase in glucose utilization in the liver organoids (Tao et al., 2022). Furthermore, under hyperglycemic conditions, the organoid appeared dysfunctional and could be treated with metformin. Hence, this multi-organoid system can recapitulate the relevant liver-islet axis in humans under physiological and pathological conditions, and also provides a unique platform for future T2DM research and drug development (Tao et al., 2022). Moreover, multicellular human liver organoids consisting of hepatocytes, stellate cells, and Kupffer-like cells have been created, which can further mimic the complexity of real liver tissue (Ouchi et al., 2019). It is worth noting that the rational use of new materials has the effect of improving drug efficacy; however, their clinical application can be hindered by possible side effects such as liver toxicity. In this regard, liver organoids can also provide rapid toxicity screening of nanomaterials and contribute to the safe use of medicines (Zhang et al., 2024).

### 2.7 Other organoids

Diabetes can cause severe damage to the endothelial cells and pericytes of blood vessels, which in turn can lead to serious complications, including DR and DN. Wimmer et al. generated blood vessel organoids from hPSCs and modeled the influence of diabetes on the vasculature, such as basement membrane thickening. They used the organoids to identify DLL4 and NOTCH3 as risk factors for diabetic vasculopathy (Wimmer et al., 2019a; Wimmer et al., 2019b). Liu et al. summarized the methods of generating blood vessel organoids and organoid vascularization, with emphasis on the roles of Notch, Wnt, BMP, VEGF, and PDGF signaling pathways in vascular differentiation (Liu C. et al., 2020). Strategies for organ-specific diabetic vascular disease models have also been established, facilitating research into diabetic vascular complications (Naderi-Meshkin et al., 2023). Additionally, to mimic human muscle insulin resistance and to study the molecules responsible for it, an ihPSC-derived myotube was also invented which had significant defects in glucose metabolism (Iovino et al., 2016). Self-assembled cardiac organoids can also be used to study cardiac development and mimic maternal diabetes-induced congenital heart disease (Lewis-Israeli et al., 2021).

## 3 The role of organoids in diabetes research

**FIGURE 3:**
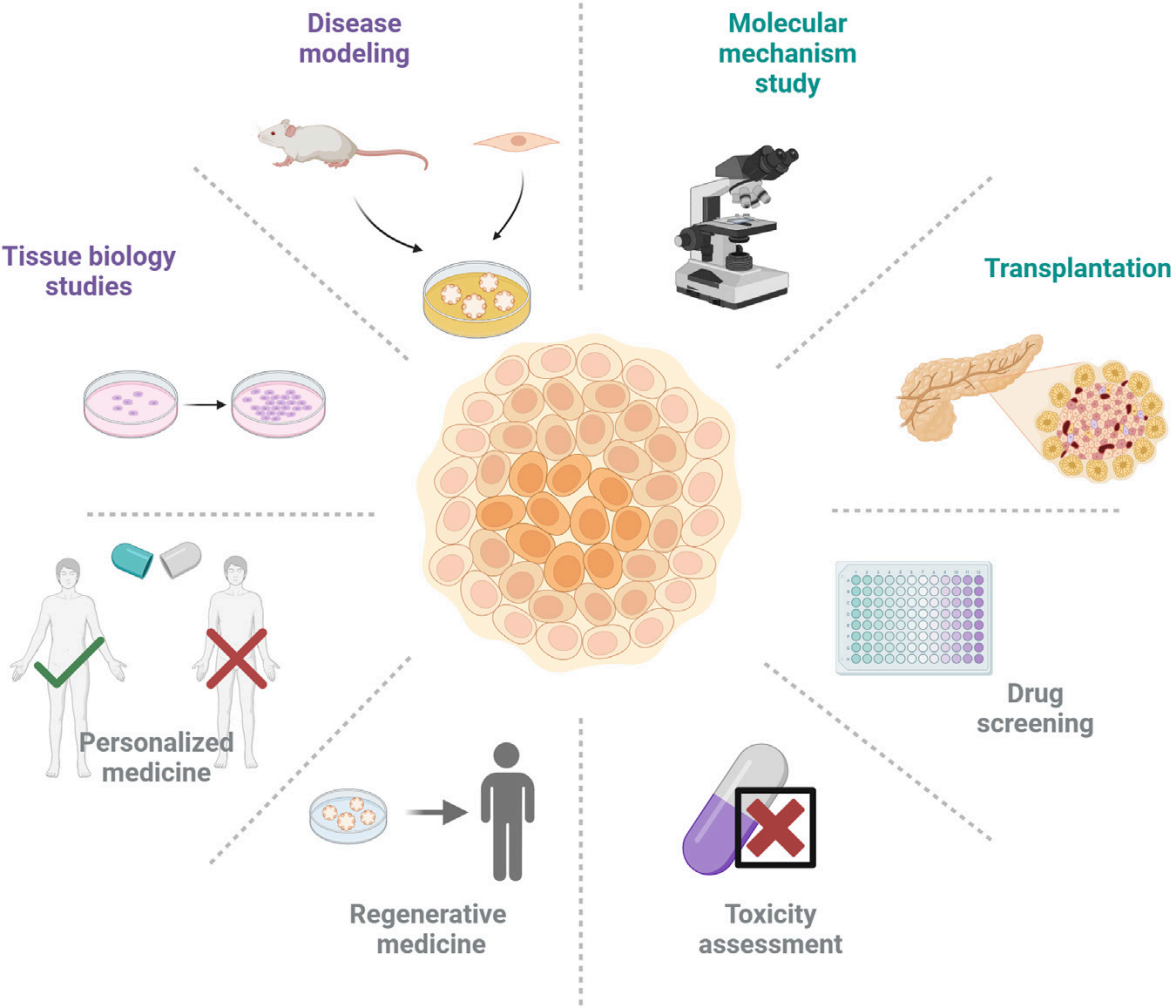
Various applications of organoid technology in the field of diabetes and its complications.

Organoids can mimic the structure and function of organs *in vitro* and have a wide range of translational applications including disease modeling, drug screening, and cell therapy (Figure 3).

### 3.1 Organoids for pathogenesis studies

Organoids are widely-used in probing the pathogenesis and factors influencing diabetes mellitus. Through validation on null intestinal organoids, it was found that inhibition of intestinal Gpr17 expression could promote GLP-1 secretion and thus improve glucose metabolism, suggesting that GPR17 may be a potential interventional target for the treatment of diabetes mellitus (Yan et al., 2022). It was also discovered that miR-144, a micro-RNA involved in post-transcriptional regulation, could target IRG2 to regulate NRF1(Azzimato et al., 2021). miR-144 plays a key role in insulin resistance, and this result was also validated in a liver organoid: by silencing miR-144, the activity of fumarate hydratase, which plays a key role in the miR-144/IRG2/NRF1 pathway, was decreased (Azzimato et al., 2021). Using human brown fat organoids, Reverte-Salisa’s research showed that EPAC1 can increase the proliferation and differentiation of brown fat cells, suggesting that EPAC1 may have the potential to increase energy expenditure and thereby combat metabolic diseases (Reverte-Salisa et al., 2024). Using liver organoids, L22RA1 was found to be closely related to liver lipid homeostasis, thereby affecting glucose tolerance and insulin resistance (Huang et al., 2025). Also, the development of human vascular organoids helps to gain a deeper understanding of the vascular effects caused by diabetes at the single-cell level and to identify diabetes-related genes (Nikolova et al., 2025).

Monogenic diabetes, including neonatal diabetes and maturity-onset diabetes of the young (MODY), is characterized by early onset. It is often caused by mutations in genes involved in pancreatic development and insulin synthesis and secretion (Aguilar-Bryan and Bryan, 2008; Bonnefond et al., 2023). However, people with T2DM can also suffer from monogenic diabetes (Bonnefond et al., 2023). Organoids are uniquely suited for research in this area, either by using patient-derived iPSCs and comparing them to CRISPR/Cas9 gene-corrected autologous cells, or by genetically modifying hPSCs to mimic the disease-related gene mutations of the patient. Based on this strategy, researchers identified and validated the roles of STAT3, NEUROG3, PDX1, ONECUT1, and MAFB in pancreatic and β-cell development and diabetes progression (Saarimäki-Vire et al., 2017; Wang et al., 2019; Russell et al., 2020; Philippi et al., 2021). They also demonstrated the mechanism of endocrine regulation by ONECUT1 in single-gene and multifactorial diabetes mellitus (Philippi et al., 2021). Endoplasmic reticulum stress is also present in these mutant phenotypes (Panova et al., 2022).

Organoids are also helpful in studying the effects of environmental factors and the body’s metabolic state on tissues and organs *in vivo*. Using intestinal organoids, researchers have validated the powerful activation of peroxisome proliferator-activated receptor δ (PPAR-δ) by a high-fat diet (Beyaz et al., 2016). To ensure normal insulin secretion, pancreatic β-cells were exposed to glucose and oxygen for a long period of time. This β-cell exposure to high glucose concentrations *in vitro* also leads to a hypoxic phenotype and activation of hypoxia-inducible factor-1α (HIF-1α), which is inextricably linked to glucose metabolism, oxidative stress, and angiogenesis (Gunton, 2020; Catrina and Zheng, 2021; Li et al., 2021). Coherently, insulin secretion-related indices improved by applying the HIF-1α inhibitor PX-478 to human islet organoids exposed to hyperglycemia (Ilegems et al., 2022). Researchers have worked to keep pancreatic progenitor cells *in vitro* close to their counterparts *in vivo*; hence, the resulting pancreatic organoids can be more accurately tested for the effects of genes that predispose to diabetes or other diseases of the pancreas (Gonçalves et al., 2021). Moreover, as adipose tissue is highly heterogeneous with many cell types (Corvera, 2021), adipose organoids are more than capable of further simulating real adipose tissue. A type of adipose spheroid sensitive to environmental changes was created to provide a system for assessing the effects of different factors on adipocytes (Klingelhutz et al., 2018).

With a deeper understanding of the molecular regulatory mechanisms of a disease, organoids can be instrumental in precision medicine and personalized therapy. Thiazolidinediones target the transcriptional activity of PPARγ to increase insulin sensitivity and reverse insulin resistance in patients with T2DM. Wenxiang Hu et al. used human adipose stem cell-derived adipocytes to demonstrate that the specific single nucleotide polymorphism rs4743771 could regulate the PPARγ target gene ABCA1, and that single nucleotide polymorphisms were enriched in the patient-specific PPARγ binding site, which correlates with the individual-specific effect of the azolidinediones drug rosiglitazone, explaining the inefficacy of this type of drug in some patients with diabetes(Nanjan et al., 2018; Hu et al., 2019). This model for studying human genetic variation in response to antidiabetic drugs may have important implications for the development of personalized therapies for metabolic disorders.

### 3.2 Organoids for drug development and screening

Organoid technology also shows the potential for the high-throughput screening (HTS) of drugs. Using hESC-derived glucose-responsive cells, mutations in CDKAL1, KCNQ1, and KCNJ11 were determined to cause impaired glucose secretion. On the basis of this, a further screen identified a drug candidate that could rescue CDKAL1-specific defects by inhibiting the FOS/JUN pathway (Zeng et al., 2016). A drug candidate, Galunisertib, was found to rescue GLIS3 mutation-induced β-cell death using a chemical screen based on a derivative of GLIS3^−/−^ hESCs (Amin et al., 2018). Functional stem cell-derived β-cells for type 1 diabetes were developed and the efficacy of the diabetes drugs toluenesulfonylurea, liraglutide, and GCK activators was validated in this model (Millman et al., 2016).

Pathological damage can be induced in hepatic organoids using oleic and palmitic acids, and these organoids can be used as a platform for drug screening to assess toxicity and efficacy. For example, the antidiabetic drug troglitazone, which was withdrawn from the market due to its severe idiopathic hepatotoxicity, showed toxicity in the organoids. Moreover, the effects of metformin could be evaluated in the organoids and showed favorable results (Mun et al., 2019).

Diabetes is a multi-organ systemic disease in which various organs and tissues interact with each other in the development of the disease. A microfluidic multi-organoid system that mimics the human liver-islet axis has been established, which allows co-culture of hiPSC-derived liver and islet organoids (Tao et al., 2022). In this organoid system, metabolism-related pathways are activated, and pancreatic islet organs can secrete insulin and further contribute to increased glucose utilization in liver organoids (Tao et al., 2022). Meanwhile, the pathological state was simulated using high glucose conditions, which showed pathological damage consistent with the body’s response. Moreover, this damage could be alleviated by metformin suggesting that this multi-organoid system can effectively mimic the liver-islet axis in either physiological or pathological states and provide a feasible solution for disease research and drug development.

At present, diabetes treatment not only requires blood glucose control but also management of vascular diseases, kidney diseases, and obesity. As mentioned earlier, GLP-1 agonists play a significant role in diabetes treatment, offering both blood glucose control and vascular protection. Pinto utilized human intestinal organoids to assess the efficacy of the GLP-1 analog semaglutide. They optimized its formulation and found that nanoparticles targeting the intestinal Fc receptor enhanced the absorption of semaglutide (Pinto et al., 2024). Qi Lin and colleagues established a microphysiological system comprising hiPSC-derived white adipocytes, hepatocytes, and macrophages, and further discovered that semaglutide can improve hepatocyte function by targeting adipocytes (Qi et al., 2024). However, the needs of some obese diabetic patients remain unsatisfied when using GLP-1 agonists alone. Tirzepatide is a dual receptor agonist for the glucose-dependent insulinotropic polypeptide (GIP) receptor and the GLP-1 receptor. Clinical studies have demonstrated that tirzepatide has good safety profiles. Compared to semaglutide, it shows better blood sugar control and weight loss effects, significantly delaying the progression of diabetes (Garvey et al., 2023; France and Syed, 2024; Aronne et al., 2025; Jastreboff et al., 2025). E Lorza-Gil and colleagues established an organoid model of pancreatic adipose tissue, whose adipogenesis marker levels are comparable to those of natural pancreatic adipocytes. This model not only effectively simulates the pancreatic fat-islet crosstalk but also retains donor-specific metabolic memory (Lorza-Gil et al., 2025). Using this organoid model to evaluate the therapeutic effects of tirzepatide, the study demonstrated that tirzepatide stimulates lipolysis and reduces levels of inflammatory factors IL-6 and MCP-1 (Lorza-Gil et al., 2025). And I believe that the application of vascular organoids and vascularized organoids will also further evaluate and validate the vascular and cardiovascular protective effects of tirzepatide.

These examples suggest that drug candidates that rescue gene-specific defects can be identified using organoid technology, paving the way for precision therapy of metabolic diseases. We also summarized organoids used in diabetes mechanism research and drug screening (Table 1).

**TABLE 1:** Organoids for diabetes mechanism research and drug screening.

Source/Methods	Organoid types	Treatment	Main research target	Validated drugs	Refs
intestinal Gpr17 knockout mice	intestinal organoid	Gpr17^−/−^	Gpr17	-	Yan et al. (2022)
co-cultures of hepatocytes and non-parenchymal cells	human liver organoids	miR-144 silencing	miR-144	-	Azzimato et al. (2021)
Patient-derived iPSCs	Differentiation of PSCs into β-like cells	-	ONECUT1	-	Philippi et al. (2021)
hiPSC	human brown fat organoids	preferential activator of EPAC1	EPAC1	-	Reverte-Salisa et al. (2024)
hiPSCs	human liver organoids	-	IL22RA1	-	Huang et al. (2025)
SVF-derived organoids	human pancreatic adipose tissue organoids	-	GLP1R/GIPR	Tirzepatide	Lorza-Gil et al. (2025)
HUES9 cells	human intestinal organoids	semaglutide was incorporated into PLGA-PEG nanoparticles	intestinal Fc receptor	Semaglutide	Pinto et al. (2024)
Patient-derived iPSCs	human islet organoids	high glucose	HIF-1α	HIF-1α inhibitor PX-478	Ilegems et al. (2022)
hESC	pancreatic organoids	CDKAL1^−/−^	CDKAL1	T5224	Zeng et al. (2016)
hESC	mono-hormonal glucose-responding pancreatic β-like cells	GLIS3^−/−^ hESC	GLIS3	Galunisertib	Amin et al. (2018)
Patient-derived hiPSCs	functional stem cell-derived β-cells	glucose	-	Sulfonylurea, liraglutide, GCK activators	Millman et al. (2016)
PSCs	human hepatic organoids	oleate and palmitate	-	Troglitazone, metformin	Mun et al. (2019)
hiPSC	Islet organoids, liver organoids	high glucose	-	metformin	Tao et al. (2022)

### 3.3 Organoids for organ transplantation and cellular replacement therapy

Cell therapy is considered a viable option in the treatment of diabetes, replenishing ß-cells and maintaining glucose homeostasis through pancreas or islet transplantation (Lysy et al., 2013). Clinically, a 68 kg person with type 1 diabetes needs about 340–750 million transplanted islet cells. Organoid technology allows for the suspension of 300 million cells in a single 500 mL flask, which undoubtedly alleviates the shortage of donors in the clinic (McCall and Shapiro, 2012; Pagliuca et al., 2014). Researchers found that introducing vitamin C at an early stage of pancreatic progenitor cell differentiation led to the generation of PDX1/NKX6.1 pancreatic progenitor cells, and that further differentiation using agents including ALK5 inhibitors, BMP receptor inhibitors, and thyroid hormone led to the upregulation of NGN3 and a population of cells a large fraction of which co-expressed PDX1, NKX6.1, NEUROD1, and NKX2.2(Rezania et al., 2014). Addition of notch inhibitors to the above leads to the generation of cell populations in which a large proportion of PDX1/NKX6.1/NEUROD1 cells express insulin (Rezania et al., 2014). These highly differentiated cells rapidly reversed diabetes after transplantation in mice (Rezania et al., 2014). Moreover, patient-derived hiPSCs can differentiate to generate PDX1+/NKX6-1+ cells, which can be transplanted into mice to spontaneously generate glucose-responsive cells (Millman et al., 2016).

Furthermore, the ability to transform terminally differentiated cells could also be a powerful tool for treating diabetes. With the help of the reprogramming factors Pdx1, MafA, and Ngn3 (PMN), researchers were able to transform intestinal crypt cells into endocrine cells, which were shown to be glucose-responsive and able to ameliorate hyperglycemia in diabetic mice. Moreover, PMN has the ability to spur the transformation of intestinal epithelial cells into beta-like cells in human intestinal-like organoids (Chen et al., 2014). Recently, Eiji Yoshihara et al. added hADSCs and HUVECs during the differentiation of hiPSC-derived endocrine progenitor cells to form multicellular spheroids (MCSs) that were comparable to the size of human pancreatic islets and contained insulin-secreting cells. MCSs transplanted into the renal capsule were able to maintain glucose homeostasis in STZ-induced NOD-SCID mice for a period of time and could regulate insulin secretion according to the state of the organism, which was also similar to human insula transplantation (Yoshihara et al., 2020). In a study by Daisong Wang et al., a novel islet progenitor cell, Procr islet cell, was identified and cultured *in vitro* to become an islet-like organoid that included all endocrine cell types (Wang D. et al., 2020). The organoid was transplanted into the kidney capsule of STZ-induced diabetic mice and showed that the organoid could secrete insulin and attenuate weight loss and hyperglycemia in mice, showing similar effects to those with fresh islet transplantation (Wang D. et al., 2020). In addition, the integration of human amniotic epithelial cells has been reported to enhance the transplantation success of pancreatic islet organoids (Lebreton et al., 2019). Interestingly, it has also been suggested that intestinal cells can be transformed by cellular reprogramming into cells that can produce insulin (Du et al., 2022). Similarly, human gastric stem cells were utilized to differentiate into islet organoids containing gastric insulin-secreting cells, and such gastric-derived human insulin-secreting organoids could effectively restore glucose homeostasis in mice. It is worth mentioning that human gastrointestinal stem cells can be easily biopsied, and can be successfully cultured and expanded *in vitro*, providing a precious resource for autologous cell therapy (Huang et al., 2023). However, secretion of islets after transplantation is still an issue to be considered, which may be related to islet size (Fujita et al., 2011; Zhang et al., 2022).

Adipocytes derived from hPSCs also provide a unique program for studying metabolic diseases. BAs are considered a potential cell source for treating metabolic diseases such as diabetes (Zhang et al., 2020) as their increased activity also contributes to insulin sensitivity in patients with diabetes (Sakers et al., 2022). Several teams have successfully induced adipocytes and found that PPARG2, CEBPB, and PRDM16 play important roles for the differentiation of mesenchymal progenitor cells derived from PSCs to white or brown adipocytes. When these induced adipocytes were transplanted into mice, they had mature morphological and functional characteristics (Ahfeldt et al., 2012). A protocol for generating BAs from hPSCs via a paraxial mesoderm intermediate shows that transplanted BAs exhibit multilocular lipid droplet morphology and elevated UCP1 expression, increased metabolism in recipient mice, reduced circulating glucose levels in hyperglycemic mice, and no tumorigenic effect (Zhang et al., 2020). It is also worth mentioning that engineered adipocytes created using CRISPR-Cas9 technology-which is best characterized by modifying white adipocytes to a BA phenotype-improved glucose tolerance and insulin sensitivity in mice after transplantation. This suggests that this means of modification may open up another unique therapeutic direction for obesity and diabetes (Wang C.-H. et al., 2020).

## 4 The role of organoids in diabetic complications

### 4.1 Diabetic retinopathy (DR)

First, it is feasible to utilize organoids for the study of pathological mechanisms of DR. In addition to the high degree of structural and functional similarity to the human retina, more importantly, the cell type specificity of disease-related gene expression present in the human retina is present in the organoid (Cowan et al., 2020). Furthermore, a study by Capowski et al. showed that ROs could be generated repeatedly from 16 hPSC lines, reducing inconsistencies between cell lines (Capowski et al., 2019). The three stages of retinal organoid growth can also be easily distinguished morphologically by optical coherence tomography (Capowski et al., 2019). Taken together, this makes it feasible to use organoids to study disease mechanisms and to undertake targeted repair of the human retina (Gagliardi et al., 2018; Miltner and Torre, 2019; Cowan et al., 2020). For example, researchers recently developed a physiologically similar 3D Outer Blood-Retinal Barrier model with choroidal capillaries (Nam et al., 2023). When simulating diabetic pathological conditions, the model shows typical features seen in patients with diabetes, such as reduced tight junctions and impaired barrier functions (Nam et al., 2023). Additionally, recent studies utilizing ROs have found that an unbalanced unfolded protein response (UPR) is associated with DR-related retinal toxicity caused by 1-deoxysphingolipids, and that ATF6 activation attenuates 1-dSL toxicity (Rosarda et al., 2023). This suggests that targeting 1-dSL and the UPR is promising for the treatment of DR (Rosarda et al., 2023). This also reveals the feasibility of utilizing ROs to study DR-related mechanisms.

Interestingly, hiPSC-derived ROs have been reported to release exosomes and microvesicles with small non-coding RNAs that have been implicated in the regulation of human retinal development (Zhou et al., 2021). Exosomes play a special and important role in many biological barriers, including the blood-brain barrier and blood retinal barrier (BRB), which may promote retinal development or accelerate the development of retinal diseases, including DR (Elliott and He, 2021). On the one hand, in DR, exosomes in platelet-rich plasma have been observed to cause retinal endothelial damage through upregulation of the TRL4 signaling pathway (Liu J. et al., 2020). On the other hand, exosomes from retinal astrocytes contain anti-angiogenic components that inhibit choroidal neovascularization, protecting the eye from angiogenesis and maintaining its functional integrity (Hajrasouliha et al., 2013).

Second, the role of ROs in drug screening and delivery can be reflected in the following aspects. Organ-on-a-chip technology combining iPSC-organoids, with the advancement of bioengineering technology and the introduction of microfluidic systems, can better mimic the *in vivo* microenvironment and facilitate long-term RO maintenance; consequently, becoming a good platform for drug development (Kutlehria and Singh, 2021; Xue et al., 2021). For example, patients with diabetes have higher expression of HIF and HIF-regulated vasoactive mediators. Using mouse models and ROs, researchers have demonstrated that the newly developed HIF inhibitor 32-134D shows promise for clinical development; that is, it can effectively inhibit HIF accumulation and regulate related gene expression, thereby preventing retinal neovascularization and increased vascular permeability, which are typical features of DR (Zhang et al., 2023). Moreover, intraocular injection of various drugs and retinal gene therapy using adeno-associated virus (AAV) delivery are effective and widely adopted therapies for the treatment of DR and many other ophthalmic diseases (Trapani and Auricchio, 2018; Yu et al., 2023). Therefore, the human retinal microarray model integrating iPSC-ROs and retinal pigment epithelial cells in a microfluidic platform has the potential to test the performance of different types of AAV vectors (Achberger et al., 2021). In addition, the human retinal microvessel chip can mimic the human BRB and thus can be used for drug discovery and to test the effect of drugs on the barrier properties of the BRB (Ragelle et al., 2020). Furthermore, extracellular vesicles (EVs) can not only load specific molecular cargoes, but also have strong targeting capabilities and the ability to cross certain biological barriers. hiPSC-derived ROs have been found to release EVs and are therefore emerging as a promising platform for drug delivery (Zhou et al., 2021). In conclusion, organoids provide a more reliable preclinical model that can be used for drug clinical translational research.

Finally, ROs have potential applications in organ transplantation and cellular replacement therapy. Given the interdependence of photoreceptors, retinal pigment epithelial cells, and the choroidal capillary complex, almost any advanced retinal disease process will eventually result in the loss of all these tissues (MacLaren et al., 2016). Therefore, cell replacement or regeneration may be required for patients who develop end-stage retinal degeneration. ROs derived from PSCs contain all major retina-specific cell types: anaplastic cells, bipolar cells, horizontal cells, retinal ganglion cells, Müllerian glial cells, rods, and cones, and even have a hierarchical structure (Jin et al., 2019; Afanasyeva et al., 2021). Thus, reliable ROs provide an adequate cell resource for transplantation. The Lancet reported the first successful transplantation of a stem cell source for human retinal disease, and the findings suggest that hESC-derived cells could provide a potentially safe new source of cells for the treatment of a variety of medical diseases requiring tissue repair (Schwartz et al., 2015). Recently, naïve hiPSCs (N-hiPSCs) have also been used in the study of DR (Park et al., 2020). Naïve diabetic vascular progenitor cells differentiated from N-hiPSCs have excellent high epigenetic plasticity and genetic stability, and can effectively migrate to damaged retinal sites, providing a viable direction for regenerative medicine (Park et al., 2020).

### 4.2 Diabetic nephropathy (DN)

Organoid technology has paved the way for the study of DN. As we all know, genetic factors may be one of the important determinants of the incidence and severity of DN, and hereditary lesions of glomerular structures may cause proteinuria (Liu et al., 2022). Using patient-derived kidney organoids, researchers found that mutations in the NPHS1 gene can cause congenital nephrotic syndrome (Tanigawa et al., 2018). Jun Wei Chan et al. verified the role of the HNF1A/SLC51B/estrone sulfate pathway in renal development and diabetogenesis using hiPSC-derived renal organoids from patients with MODY (Chan et al., 2023). Diabetic renal fibrosis is the basic pathological feature of DN. The degree of fibrosis is an important marker of the progression of DN, from diffuse thickening of the glomerular basement membrane in the early stage to glomerulosclerosis or tubular atrophy in the late stage (Ibrahim and Hostetter, 1997; Bloomgarden, 2005; Xiang et al., 2020). Xiaoping Yang and colleagues found that bile acid receptor agonists could reverse TGF-β1-induced renal fibrosis by regulating the Farnesoid X receptor, p-SMAD3, and TAZ in iPSC-derived renal organoids, demonstrating that bile acid receptor agonism exists in the early stage of renal fibrosis (Yang et al., 2024). Elsewhere, organoids could also shed new light on how diabetes leads to renal susceptibility to COVID-19 (Xia et al., 2022). The renin-angiotensin-aldosterone system plays an important role in blood pressure regulation, and the action of angiotensin II on the AT one receptor promotes vasoconstriction, which in turn has additional effects on renal blood flow and glomerular capillary pressure (Bloomgarden, 2005). The use of renal organoids has revealed that ACE2 increases the susceptibility of patients with diabetes to COVID-19 infection (Garreta et al., 2022).

Regarding drug screening and toxicity testing, organoids are potentially powerful tools for HTS. A fully automated HTS-compatible organoid platform has been developed for drug safety and efficacy prediction (Czerniecki et al., 2018). The kidney is an important metabolic organ of the body and kidney organoids have potential for toxicity testing and assessment (Fritsche et al., 2021). Moreover, experts suggest that new therapies currently in clinical trials for chronic kidney disease, including dual angiotensin receptor/endothelin receptor blockers and sodium-glucose cotransporter two inhibitors, can also be designed and optimized using organoids (Ramos et al., 2020).

## 5 Conclusion and future perspectives

Organoids are derived from ESCs and iPSCs from healthy individuals or patients, as well as organ-restricted ASCs, which can be cultured *in vitro* to build 3D structures and mimic the cellular heterogeneity, structure, and function of human organs. In recent decades, researchers have successfully generated various types of organoids and utilized organoid technology for disease modeling and drug screening. More importantly, Organoids have been found to have great clinical therapeutic potential. Consequently, we reviewed the progress of organoid application in diabetes and its complications, and demonstrated the broad potential of organoid technology in disease research, aiming to provide a theoretical basis and methodology for the study of related diseases.

Currently, there are several common strategies for modeling various diseases using organoid technologies. One involves the use of patient-derived hiPSCs and CRISPR-CAS technology for modeling various genetic diseases and exploring the role of a specific gene in disease progression. Another involves modelling environmentally induced acquired pathologies by exposing organoids to media supplemented with specific inducing factors or by simulating disease-inducing environments (e.g., hyperglycemic or hypoxic environments). Moreover, it is also possible to explore the driving factors of various pathologies in the human body and to screen for drivers that either ameliorate or exacerbate the phenotype of a disease (Beydag-Tasöz et al., 2023).

**FIGURE 4:**
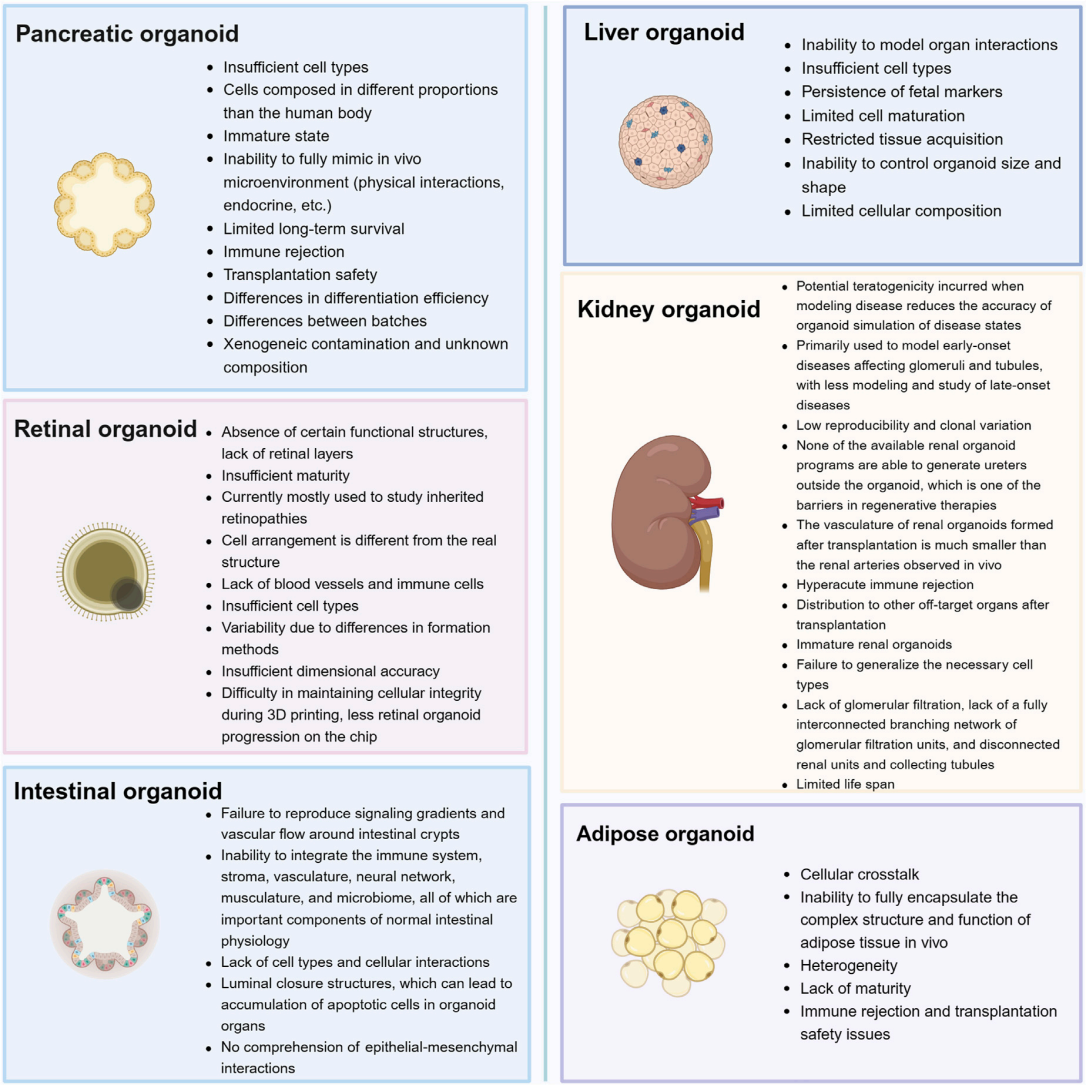
Current challenges and perspectives for organoids that can be used in the study of diabetes and its complications.

Although organoid technology with its unique innovation and creativity is rapidly evolving, and many types of organoids have promising applications in the field of research on diabetes and its complications, there are still several obstacles that need to be addressed. Therefore, we also summarized the current limitations and challenges of organoids technology (Figure 4), which may help indicate a feasible direction for better development of organoids.

First, there is still a gap between organ tissues and natural organs, which does not faithfully reflect the cellular diversity of the original tissues or the crosstalk and influence between different organs in an organism. Hence, reproducibility may be poor. Moreover, high intrinsic variability may mask therapeutic effects when validating drug action (Rossi et al., 2018; Bock et al., 2021). For example, when studying diseases, immune and inflammatory factors play a significant role, whereas organoid tissues often do not contain immune cell types (Richards et al., 2020).

Second, some organoid models tend to more closely resemble the state of the organ at the fetal stage (Shinnawi et al., 2019); hence, they can only simulate early-onset disease or the early stages of disease. During organoid culture, organoids lacking a vascular system may cause apoptosis or even tissue cavities due to hypoxia and nutrient deficiency (Fantin et al., 2013; Giandomenico et al., 2019). There have been some efforts to overcome these obstacles, including the use of small molecule compounds (e.g., BDNF) to precondition the organoids, which can develop over a longer period (Quadrato et al., 2017). Moreover, the teratogenicity that may be introduced when modeling the environment in which the disease occurs should be considered. Investigations are need as to whether this interferes with the screening of disease targets and shortens the lifespan of the organoid, which is itself one of the limitations of organoids.

To make up for these deficiencies, many vascularization strategies have been developed, which can well prolong the life span of organoids, promote tissue maturation, and simulate the microenvironment of the organoids so as to make them closer to the real situation. Currently, the commonly used vascularization strategies are also divided into two types: *in vitro* and *in vivo*. *In vitro* methods include co-culturing organoids with endothelial cells, while *in vivo* methods mainly involve transplanting organoids into immunodeficient mice. Various studies have shown that transplanted organoids can be effectively vascularized and connected to the host mouse circulatory system. It is worth mentioning that more vascularization methods, such as microfluidic devices, have been developed by combining them with bioengineering and nanotechnology (Matsui et al., 2021).

There are many more modeling methods to think about and improve, and more organoid models need to be created. Some of the organoid models presented in this review are mostly used to simulate spontaneous diseases. This begs the questions: how can we better simulate acquired diseases? Can glomerular filtration limitation be realized using bioengineering techniques? It is believed that with further development of assembly and vascularization techniques, organoids will gradually mature and improve. In a nutshell, the 3D organoid system greatly complements the existing modeling system and may play a significant role in future basic and clinical research.

## Abbreviations

ES, embryonic; iPSC, induced pluripotent stem cell; ASC, adult stem cell; RO, retinal organoid; 3D, three-dimensional; DR, diabetic retinopathy; DN, diabetic nephropathy; EGF, epidermal growth factor; hPSC, human pluripotent stem cell; ECM, extracellular matrix; HUVEC, human umbilical vein endothelial cells; MSC, mesenchymal stem cell; RPC, retinal progenitor cell; IGF1, insulin-like growth factor 1; ACE2, angiotensin-converting enzyme 2; GLP-1, glucagon-like peptide-1; T2DM, type 2 diabetes mellitus; BA, brown adipocyte; PPARγ, peroxisome proliferator active receptor γ; CEBPB, CCAAT-enhancer-binding protein-β; MODY, maturity-onset diabetes of the young; HIF-1α, hypoxia-inducible fator-1α; HTS, high-throughput screening; MSC, multicellular spheroid; UPR, unfolded protein response; BRB, blood retinal barrier; AAV, adeno-associated virus; EV, extracellular vesicle.
